# Premature or Small for Gestational Age Discrimination: International Multicenter Trial Protocol for Classification of the Low-Birth-Weight Newborn Through the Optical Properties of the Skin

**DOI:** 10.2196/16477

**Published:** 2020-07-14

**Authors:** Zilma Reis, Gabriela Vitral, Rodney Guimarães, Juliano Gaspar, Enrico Colosimo, Sergio Taunde, Nilza Mussagy, Rita Rosado Santos, Diogo Ayres-De-Campos, Roberta Romanelli

**Affiliations:** 1 Faculty of Medicine, Universidade Federal de Minas Gerais, Brazil Belo Horizonte Brazil; 2 Statistics Department, Universidade Federal de Minas Gerais Belo Horizonte Brazil; 3 Hospital Central de Maputo Maputo Mozambique; 4 Faculty of Medicine Universidade de Lisboa Lisbon Portugal

**Keywords:** prematurity, fetal growth restriction, childbirth, skin physiological phenomena, photomedicine, equipment and supplies

## Abstract

**Background:**

A low birth weight is an independent risk factor for adverse infant outcomes and a predictor of chronic disease in adulthood. In these situations, differentiating between prematurity and small for gestational age (SGA) or simultaneous conditions is essential to ensuring adequate care. Such diagnoses, however, depend on reliable pregnancy dating, which can be challenging in developing countries. A new medical optoelectronic device was developed to estimate gestational age (GA) at birth based on newborn skin reflection.

**Objective:**

This study will aim to evaluate the device’s ability to detect prematurity or SGA, or both conditions simultaneously as well as predict short-term pulmonary complications in a cohort of low-birth-weight newborns.

**Methods:**

This study protocol was designed for a multicenter cohort including referral hospitals in Brazil and Mozambique. Newborns weighing 500-2500 g will be eligible for inclusion with the best GA available, considering the limited resources of low-income countries. Comparator-GA is based on reliable last menstrual period dating or ultrasound assessment before 24 weeks’ gestation. Estimated GA at birth (Test-GA) will be calculated by applying a novel optoelectronic device to the newborn’s skin over the sole. The average difference between Test-GA and Comparator-GA will be analyzed, as will the percentage of newborns who are correctly diagnosed as preterm or SGA. In addition, in a nested case–control study, the accuracy of skin reflection in the prediction of prematurity-related respiratory problems will be evaluated. The estimated required sample size is 298 newborns.

**Results:**

Teams of health professionals were trained, and standard operating procedures were developed following the good practice guidelines for the clinical investigation of medical devices for human participants. The first recruitment started in March 2019 in Brazil. Data collection is planned to end in December 2020, and the results should be available in March 2021.

**Conclusions:**

The results of this clinical study have the potential to validate a new device to easily assess postnatal GA, supporting SGA identification when pregnancy dating is unreliable or unknown.

**Trial Registration:**

ReBec: RBR-33rnjf; http://www.ensaiosclinicos.gov.br/rg/RBR-33rnjf/

**International Registered Report Identifier (IRRID):**

DERR1-10.2196/16477

## Introduction

### Background

Low birth weight is associated with short- and long-term mortality and morbidity and is a predictor of chronic diseases in adulthood [1,2]. Small for gestational age (SGA) refers to a newborn with birth weight below the tenth percentile for gestational age (GA) and sex according to the expected standard growth curve [2]. Distinguishing between prematurity and SGA or both conditions in low-birth-weight newborns is critical to ensuring appropriate provision of care and has the potential to save lives [3,4]. Treatment of prematurity usually involves respiratory support and the administration of exogenous surfactant, to increase alveolar surface tension. Besides, most SGA newborns may have suffered from chronic intrauterine hypoxia due to placental oxygen delivery failure, and thus require specific support [5]. The diagnoses of prematurity and SGA depend heavily on a reliable GA estimation [6]. Although easy access to early obstetric ultrasonography has overcome many of the uncertainties related to pregnancy dating based on a woman’s recollection of her last menstrual period [6,7], this practice is not widely available in low- and middle-income countries [8]. Pregnancy dating based on the last menstrual period is affected by memory recall, confusion caused by early pregnancy bleeding interpreted as menstruation, irregular menses, the effect of hormonal contraception and intrauterine devices, maternal chronic disease, and poor nutrition [9,10]. Apart from this, unequal access to technological solutions in health care challenges the agenda of sustainable development goals [11] and contributes to increased neonatal morbidity and mortality. The availability of an affordable and low-maintenance device that can accurately evaluate GA at birth has the potential to rationalize management decisions and avoid unnecessary interventions and their related costs, essential aspects of health care systems [12].

### Prior Work

The optical properties of the skin’s interaction with light can be evaluated by devices that emit and receive photons [13]. Variations in light scattering through the skin are believed to be associated with changes in skin thickness, the concentration of chromophores, and light wavelength [14]. A novel technology was developed to estimate GA at birth (Test-GA) by analyzing the photobiological properties of the newborn’s skin in combination with clinical variables [15]. This noninvasive and nonionizing technology features a probe containing light emitters and receivers that is brought in contact with the skin over the sole. A mathematical algorithm provides an estimated GA within seconds, combining the skin reflection with clinical adjusters, such as birth weight and incubator stay [15].

**Figure 1:**
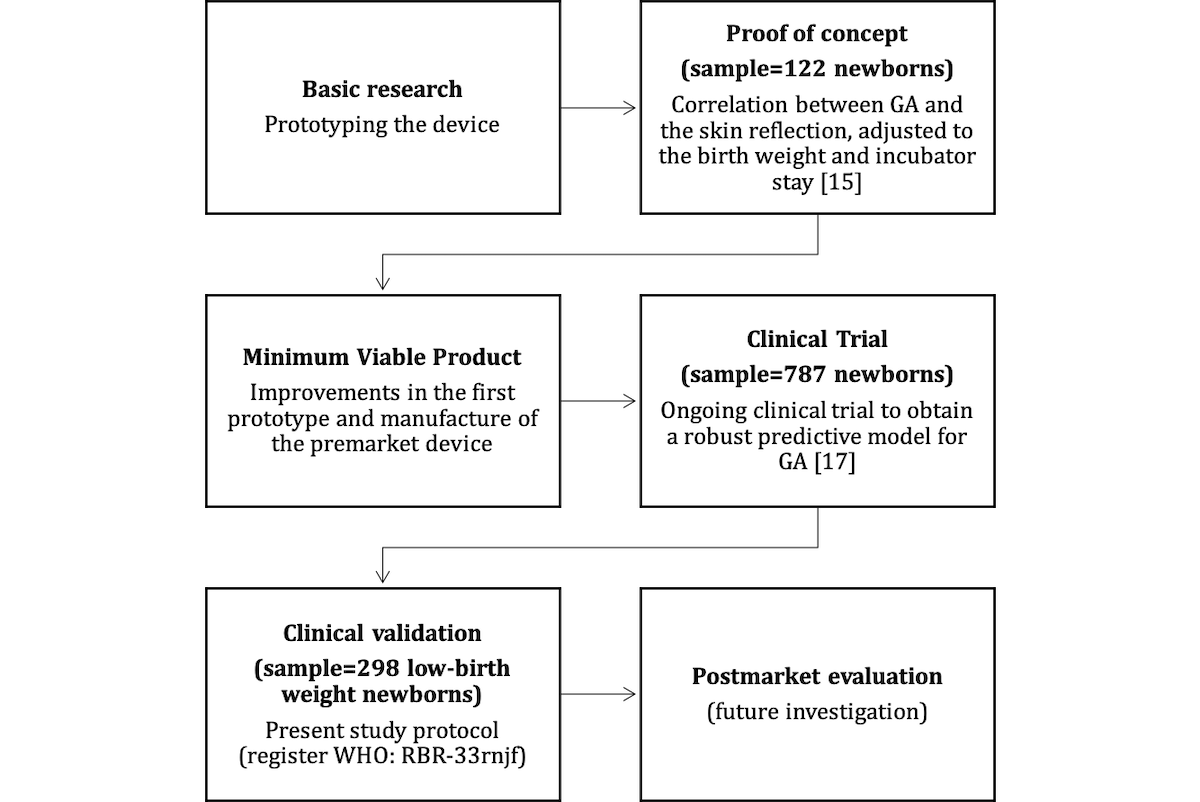
The development timeline of the preemie-test device. GA: gestational age.

The timeline of the new medical device development involves sequential steps [16], represented in Figure 1. The prototype of the device previously evaluated 115 newborns at 24-41 weeks’ gestation with promising results [15]. After insights from this first clinical experiment published in 2017, the technology received improvements to mitigate external influences in the test, such as humidity, ambient light, and to ease the user handling. The current version is a small device, about the size of a pen, with an automated ability to measure skin reflection. To reduce examiner influence, the device now warns about errors of measurement. Clinical variables are presented in a digital screen immediately after the newborn skin assessment. Since February 2019, a clinical trial is taking place in 5 Brazilian hospitals to advance the GA prediction modeling, using a sample of 787 newborns [17]. The planned date of last enrollment is July 2020.

### Present Protocol of Research

This protocol gathers data from Brazil and Mozambique referral centers. This step represents the clinical validation of the prediction model established in the preceding clinical trials. It aims to validate an optimized version of the device in low-resource birth settings, using a data set obtained in different populations, before introducing it in the market. The analysis relies on real-world antenatal GA estimations obtained during pregnancy in which first-trimester ultrasound may not always be available or reliable. The primary hypothesis of this study is that photobiological properties of the skin measured by the device, and adjusted by clinical variables, will allow the accurate prediction of GA (Test-GA) in low-birth-weight newborns.

The primary objective of this study is to validate the preemie test statistical model for GA estimation at birth and its accuracy to detect prematurity in low-birth-weight newborns. The secondary objective is to evaluate whether there is an association between skin reflection and the occurrence of early respiratory complications.

## Methods

### Study Design

This protocol describes a multicenter prospective cohort study that aims to evaluate the ability of the preemie test device to estimate GA of low-birth-weight newborns. A nested case–control study will evaluate the relationship between the optical properties of the skin and newborn respiratory complications related to prematurity.

### Study Settings, Ethics, and Dissemination

The study will be performed in the following hospitals: Hospital das Clínicas, Universidade Federal de Minas Gerais, and Hospital Sofia Feldman in Brazil; and in the Hospital Central de Maputo and Eduardo Mondlane University in Mozambique, all of which are referral centers for high-risk pregnancies and neonatal care. The University Research Council in Brazil acts as the coordinating center of this multicenter cooperation. Each local ethics review board will independently approve the study protocol. The ethical approval number is CAAE 91134218.4.0000.5149 in Brazil, and IRB00002657 in Mozambique. Parents will be asked to sign an informed consent form on behalf of the newborns as recommended by the Regulatory Bodies for Good Clinical Research Practice. The parents will retain the right to discontinue study participation at all times. The protocol of this clinical study is registered in the International Clinical Trials Registry Platform (RBR-33rnjf).

### Data Sharing Statement

The authors intend to share the minimal anonymized data set necessary to replicate the study findings. Unidentified data and study-related documents will be accessible online to the researchers and regulatory agencies. The corresponding author will provide data access under reasonable request.

### Patient and Public Involvement

A flyer with information on the relevance, aims, and procedures of this investigation will be distributed to parents of the newborns. Results will be disseminated through scientific publications, congress participation, and on the project website (http://skinage.medicina.ufmg.br). All participants will be volunteers and will not receive any compensation or advantages.

### Eligibility Criteria and Participant Timeline

A sequential enrollment of newborns will follow the inclusion of participants based on eligibility criteria until the sample size is achieved. The inclusion criteria are liveborn infants weighing 500-2499 g with GA information obtained using a qualified last menstrual period or obstetric ultrasound <24 weeks’ gestation (Comparator-GA). Exclusion criteria are structural skin alterations or conditions that modify the skin, such as anhydramnios, hydrops, congenital skin diseases, and chorioamnionitis.

Table 1 illustrates the schedule of enrollment, test application, and endpoint measurement. The skin assessment will occur during the first 24 hours of age. The newborns will be followed up for 72 hours until discharge, held in hospital (for treatment-related purposes), or death, whichever occurs first, considered the study exit. The newborns will continue to receive standard assistance after the study follow-up according to settings, resources, and local staff definitions, whether during hospital admission or at hospital discharge or re-admission.

**Table 1:** Participant timeline of the study.

Study procedures	Before Test-GA	Test-GA, ≤24 hours old	72 hours old: discharged, held in hospital, or death
Enrollment 1: Prospective cohort study^a,b^	X		
Informed consent	X		
Taking of obstetric history	X		
Intervention: Test-GA		X	
Comparator: Best estimation of GA available		X	
Enrollment 2: Nested case–control study^c^			X
Assessments and analysis^d^			X

^a^Inclusion criteria are liveborn, Comparator-GA (reliable last menstrual period [18] or ultrasound assessment at <24 weeks’ gestation [8]), birth weight (500-2499 g), and ≤24 hours old.

^b^Exclusion criteria are malformation with structural skin alterations and skin modifiers (eg, anhydramnios, hydrops, congenital skin diseases, chorioamnionitis).

^c^To determine eligibility criteria for paired allocation according to birth weight range to either the case group (TTN or RDS) or the control group (without TTN, RDS, tachypnea due to other reasons, or bloodstream infection).

^d^Neonatal outcomes including respiratory complications due to prematurity, neonatal intensive care unit admission, or ventilatory support

GA: gestational age; RDS: respiratory distress syndrome; TTN: transitory tachypnea of the newborn.

Within the study cohort, a secondary nested case–control study will include newborns with the following inclusion criteria: (1) for the case group, respiratory distress syndrome or transitory tachypnea of the newborn; (2) for the control group, newborns will be randomly selected with no respiratory diseases or manifestations by other conditions, paired by birth weight range: <1000 g, 1000-1499 g, or 1500-2499 g.

### Intervention

The assessment with the device will occur as soon as possible after birth according to the detailed protocol of the skin assessment available online [19]. The device provides automatic data acquisition with minimum operator influence and stores values in an electronic database. No values will be displayed on the device screen, thereby blinding the users to the results obtained at the time of acquisition. Characteristics of the components, wavelength of the light emitter, and external acquisition for the metering process categorized the safety level of this medical device as Class II (noninvasive and medium risk) according to the regulatory agency in Brazil. The prototype unit of measurement and process of GA estimation are patented (Nos BR1020170235688 and CTIT-PN862, respectively) [20].

### Training, Roles, and Monitoring

Teams of health professionals were trained and standard operating procedures were developed following the good practice guidelines for the clinical investigation of medical devices for human participants according to the International Organization for Standardization 14155:2011 [21] and the regulatory health agency’s recommendations for the approval of medical devices [22]. The training occurred in September 2018 in Brazilian hospitals and in July 2019 in the Hospital Central de Maputo, Mozambique. Data collection and monitoring have been happening since then. All participating centers were visited by the study coordinator to qualify the clinical investigators for training in Good Clinical Research Practice, with attention paid to the requirements for validation of medical devices and the completion of at least 30 simulated examinations. Local research coordinators contributed to development of the study protocol and will supervise the data collection and record confidentiality.

### Data Definition and Collection

Comparator-GA is calculated by obstetric data obtained at the time of enrollment. The requirements for establishing the best GA calculation (assessed by interviewing the mother) are based on the following:

- reliable last menstrual period dating, including certainty about last menstrual dating, regular menstrual cycles, no contraceptives within 3 months of conception, and no abortion or delivery 2 months prior to conception [18]; OR
- ultrasound <24 weeks’ gestation, considered reference for low-income countries as recommended by the World Health Organization (WHO) [8], even if the clinical information on last menstrual period is absent.

For data curation, pregnancy dating based on ultrasonography reports will be adjusted to Intergrowth 21 standards. Fetal crown–rump length [23] as well as femur length and head circumference will be used to recalculate GA [24]. When both ultrasound and reliable last menstrual period dating are available, correction will depend on whether there are discrepancies in excess of 5, 7, 10, or 14 days according to the identified GA at first assessment as recommended in ACOG Committee Opinion guideline number 700 (May 2017) [6].

Test-GA will be determined by statistical analysis according to signals stored in the device’s processor and clinical data. For this, the GA modeling prediction is based on the result of another ongoing clinical trial (registration number RBR-3f5bm5). This study is taking place at 5 Brazilian centers with the analysis of the skin reflection and clinical data of 787 newborns, with 24-42 weeks of gestation, using strict rules of the crown–rump length ultrasound measurement for pregnancy dating [17]. We expect to establish the prediction model toward the end of the RBR-3f5bm5 clinical trial in September 2020.

Birth weight is the first measurement performed by the local staff during the first 24 hours of life. A digital scale for standardizing weighing is already present in all settings.

A double system of clinical data collection was implemented. Trained researchers will fill out paper-based formularies and an electronic interface. Original ultrasound and clinical information reports of the antenatal care will be scanned. A double-check data conference will adjust typos comparing original paper–based documents with the electronic clinical records before the statistical analysis. The system has been designed to avoid inconsistencies in typing data according to preset constraints of expected information. Recordkeeping and server backup will be performed according to informatics best practices. To guarantee anonymity, patient identification will only be accessible by the collaborating center coordinators. The data entry form is available in Multimedia Appendix 1.

### Study Outcomes

#### Primary Outcomes

The primary endpoints will be differences between the Test-GA and Comparator-GA estimates and proportion of correct preterm newborn detections by the Test-GA at <37 weeks’ gestation with a 1-week margin of error.

#### Secondary Outcomes

The secondary endpoint will be the proportion of correctly identified SGA newborns under the tenth percentile of GA according to Intergrowth 21 standards [25]. For this endpoint, the proportion of SGA newborns detected by the Test-GA will be compared with that detected by the Comparator-GA.

Others secondary endpoints in the case–control nested study will be the capacity of the Test-GA results to predict the following neonatal complications:

- Respiratory distress syndrome based on clinical and radiological findings and respiratory outcomes [26,27].
- Transitory tachypnea of the newborn based on clinical findings and respiratory outcomes [26].
- Ventilatory support due to pulmonary immaturity.
- Neonatal intensive care unit admission for pulmonary immaturity.

### Sample Size

The sample size calculation is estimated based on the primary endpoint. Previous studies reported that Test-GA has 90%-100% accuracy at identifying premature newborns [28]. Using the inferior value of the confidence interval, an overall sample size of 298 newborns, including premature and term, is required for a robust evaluation of the device’s accuracy as recommended by Flahault et al [29].

### Statistical Analysis

To assess differences between the Test-GA and Comparator-GA estimates, average difference (with standard deviations), intraclass correlation coefficient, paired *t*-testing, and Bland–Altman scatter plots [30] will be used. To evaluate the accuracy of Test-GA in identifying preterm and SGA neonates, sensitivity, specificity (with 95% confidence intervals), and receiver operating characteristic curves and other discriminant analysis techniques will be calculated. The relationship between the measurement of the newborn’s skin reflectance and newborn’s respiratory complications due to immaturity will be evaluated using receiver operating characteristic curves, association tests, and risk ratios. The significance level for hypothesis tests will be 5%, together with 95% confidence intervals.

## Results

The study began in November 2018 with the training of health professionals in the different participating centers. Then, standard operating procedures were developed following the good practice guidelines for the clinical investigation of medical devices for human participants. The first recruitment started in March 2019 in Brazil. Data collection is planned to end in December 2020, and the results should be available in March 2021.

## Discussion

### Strengths and Limitations

The novel device is expected to contribute to the risk evaluation of the newborn, adding clinical value anywhere a child is born without GA information, with focus on scenarios with a limited access to high-cost technology. GA is a trigger information in birth scenario for making decisions on the best delivery of care [6-8]. Actions for the preterm birth morbidity and mortality mitigation face the faith that GA estimate is a trivial task [31]. This study will provide evidence of the accuracy of an original method for GA calculation at birth that will be useful in settings where GA is not routinely assessed by ultrasound during pregnancy [8]. Last menstrual period dating will be estimated by interview [18] and clinical document scanning to overcome the issue of absent ultrasound dating and memory recall inaccuracies. WHO recognizes 4-As medical devices as essential to the reliable functioning of health systems. For this proposal, the framework of development gathers Available, Accessible, Appropriate, and Affordable health technologies [32]. Effective and low-cost medical devices might be a contribution to mitigating the gap between well-equipped care settings and the needy ones for the reduction of preventable newborn deaths [8,33]. The emergent technology we test in this clinical study (ie, light scan to determine skin age) is included in the WHO compendium of innovative health technologies for low-resource settings due to the potential to improve health systems in these settings [33].

Regarding the step for a new technology validation, previous reports suggested alternative approaches for postnatal GA prediction, as well as for forecasting respiratory morbidity related to preterm birth prognosis. Using some elements of the newborn screening of congenital diseases associated with birth weight, a predicting mathematical model estimated the GA with high accuracy [34]. The accuracy analyses were useful to evaluate the performance of quantitative ultrasound texture assessment in order to predict neonatal respiratory morbidity [35].

This study will also evaluate an additional step beyond the prematurity identification based on the GA, the capacity of the technology to identify immediate respiratory complications due to immaturity in the newborn. The lung maturity of a newborn is related to a deficiency of surfactant, a phospholipid essential for alveolar stability, which is affected by the chronology of gestation and factors such as maternal disease [36]. This hypothesis is based on the synchronous development of fetal organs and tissues during gestation. We expect limitations in respiratory outcome evaluations, from the chest X-ray pattern and the improvement after surfactant therapy, as these resources are not always available in low-income settings. However, we believe that GA assessed by the new device could also help to identify those newborns who need respiratory support in the target population.

The results of this clinical study have the potential to validate a new device to easily assess postnatal GA, supporting SGA identification when pregnancy dating is unreliable or unknown. The quality of information about the chronology of pregnancy at birth is critical for the detection of premature and SGA newborns, differentiating small and sick infants from those who are small but healthy [6].

## Appendix

Multimedia Appendix 1 Data entry form.

## Abbreviations

GA: gestational age
RDS: respiratory distress syndrome
SGA: small for gestational age
TTN: transitory tachypnea of the newborn
WHO: World Health Organization
