# Supplementary material for: Premature or Small for Gestational Age Discrimination: International Multicenter Trial Protocol for Classification of the Low-Birth-Weight Newborn Through the Optical Properties of the Skin
Source: JMIR Res Protoc. 2020 Jul 14;9(7):e16477. doi: 10.2196/16477 (PMC7388049; doi:10.2196/16477)
Supplement: Multimedia Appendix 1 [file resprot_v9i7e16477_app1.docx]

**Multimedia Appendix 1 - Data entry form**

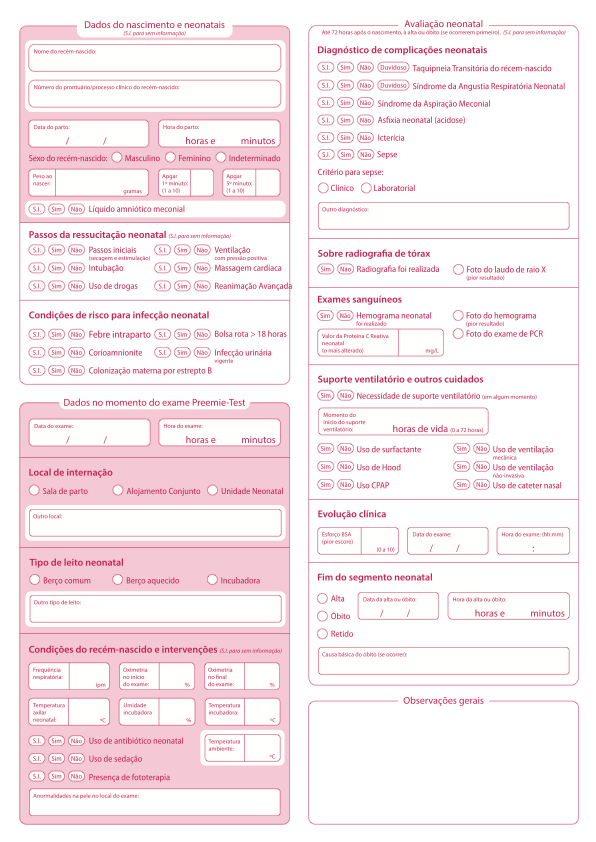


| **Entry** | **Structured options of response** |
| --- | --- |
| **Hospital Identification** | Brazil – Hospital Sofia Feldman (Belo Horizonte, Brazil)  Brazil – Hospital das Clínicas (Belo Horizonte, Brazil)  Mozambique – Hospital Central de Maputo (Maputo, Mozambique) |
| **Researcher Identification** | Name |
| **Eligibility Criteria** |  |
| Inclusion criteria | Birth < 24 hours  Antenatal ultrasound, 22 weeks of gestation  Last menstrual period available  Exclusion: Anidramnios, hydropsia, intrauterine infection |
| **Clinical Report** |  |
| **Last menstrual period** | Day-month-year |
| **Are you sure about your last menstrual period?** | 1 - Yes  0 - No |
| **Are your menstrual cycles irregulars?** | 1 - Yes  0 - No |
| **Did the conception occur within 2 months of stopping hormonal contraceptives or IUD?** | 1 - Yes  0 - No |
| **Did the conception occur within 2 months after abortion or birth?** | 1 - Yes  0 - No |
| **Gestational age at the first prenatal care visit (weeks)** |  |
| **Any disease during this pregnancy?** | 1 - Yes  0 - No |
| **Diabetes** | 1 - Yes  0 - No |
| **Hypertensive disorders** | 1 - Yes  0 - No |
| **Fetal malformation (major)** | 1 - Yes  0 - No |
| **Oligodrâmnio** | 1 - Yes  0 - No |
| **Multiple pregnancy** | 1 - Yes  0 - No |
| **Order of delivery, in case of multiple pregnancy** |  |
| **Other fetal or maternal disease** |  |
| **Antenatal Corticosteroid Therapy for Fetal Maturation** | 1 - Yes  0 - No |
| **Corticosteroid therapy scheme** | 1- Betamethasone 2 doses  2- Dexamethasone 4 doses |
| **Number of doses (Corticosteroid therapy scheme)** |  |
| **Ultrasound (US)** |  |
| **Date** | Day-month-year |
| **Gestational age (weeks)** |  |
| **Gestational age (days)** | 0-6 |
| **Crown rump length measurement (mm)** |  |
| **Biparietal diameter (cm)** | nn,n |
| **Femur length (cm)** | nn,n |
| **Head circumference (cm)** | nn,n |
| **Abdominal circumference (cm)** | nn,n |
| **Antenatal infections** |  |
| **Arbovirus (Zika, Dengue, Chikungunya)** | 1 - Yes  0 - No |
| **Cytomegalovirus (CMV)** | 1 - Yes  0 - No |
| **Enteroviruses** | 1 - Yes  0 - No |
| **HIV** | 1 - Yes  0 - No |
| **Herpes** | 1 - Yes  0 - No |
| **Parvovirus** | 1 - Yes  0 - No |
| **Varicella** | 1 - Yes  0 - No |
| **Rubella** | 1 - Yes  0 - No |
| **Syphilis** | 1 - Yes  0 - No |
| **Toxoplasmosis** | 1 - Yes  0 - No |
| **Others** |  |
| **Birth data** |  |
| **Date of delivery** | Day-month-year |
| **Delivery time** | 24 hours |
| **Sex** | 1 - Male  2 - Female  3 - Undetermined |
| **Birth weight** |  |
| **Apgar 1 minute** | 0 - 10 |
| **Apgar 5 minute** | 0 - 10 |
| **Resuscitation at birth setting** |  |
| **Steps of neonatal resuscitation:** |  |
| **Initial steps (drying and stimulation)** | 1 - Yes  0 - No |
| **Positive pressure ventilation** | 1 - Yes  0 - No |
| **Intubation** | 1 - Yes  0 - No |
| **Cardiac massage** | 1 - Yes  0 - No |
| **Drugs** | 1 - Yes  0 - No |
| **Hypoxia** |  |
| **Signs of intrapartum hypoxia:** |  |
| **Neonatal seizures** | 1 - Yes  0 - No |
| **Neonatal acidosis** | 1 - Yes  0 - No |
| **Death due to intrapartum hypoxia** | 1 - Yes  0 - No |
| **Preemie-test (GA-Test)** |  |
| **Local** |  |
| **Date of the assessment** | Day-month-year |
| **Time of the assessment** | Hour: minute |
| **Newborn’ accommodation** | 1 – Birth setting  2 – Ward with the mother  3 – Neonatal Intensive Care Unit |
| **Bed** | 1 - Cradle  2 - Heated cot  3 - Incubator |
| **Skin abnormalities during the test?** | 1 - Yes  0 - No |
| **Neonatal parameters during the assessment** |  |
| **Respiratory frequency (ipm)** | nnn |
| **Neonatal pulse-oximetry (before Preemie-test)** |  |
| **Neonatal pulse-oximetry (after Preemie-test)** |  |
| **Axillar temperature** | nnn.n |
| **Temperature inside the incubator** | nnn.n |
| **Humidity inside the incubator** | nn |
| **Ambient temperature** | nnn.n |
| **Interventions during the newborn’s follow-up** |  |
| **Antibiotics** | 1 - Yes  0 - No |
| **Sedation** | 1 - Yes  0 - No |
| **Phototherapy** | 1 - Yes  0 - No |
| **The preemie-test device** |  |
| **Signs of malfunction** |  |
| **Equipment does not load** | 1 - Yes  0 - No |
| **Empty display** | 1 - Yes  0 - No |
| **The trigger is not working** | 1 - Yes  0 - No |
| **Power led does not light** | 1 - Yes  0 - No |
| **Equipment heated up** | 1 - Yes  0 - No |
| **Skin marks or irritation after testing** | 1 - Yes  0 - No |
| **Electric shock** | 1 - Yes  0 - No |
| **Other warning event** |  |
| **72 hours – follow-up** |  |
| **Respiratory Issues** |  |
| **Transient Tachypnea of the Newborn** |  |
| **Respiratory Distress Syndrome** |  |
| **Jaundice** |  |
| **Sepsis** |  |
| **Criteria for sepsis diagnosis** |  |
| **Other neonatal complication** |  |
| **Complementary exams** |  |
| **Pulmonary X-ray** | 1 - Yes  0 - No |
| **Complete blood count** | 1 - Yes  0 - No |
| **C Reactive Protein (the one with the most altered value)** | nnn.n |
| **Ventilatory Support** |  |
| **Was ventilatory support needed?** | 1 - Yes  0 - No |
| **When the ventilatory support started?** | 0 - 72 |
| **Nasal Catheter** | 1 - Yes  0 - No |
| **Hood** | 1 - Yes  0 - No |
| **Non-invasive ventilation** | 1 - Yes  0 - No |
| **CPAP** | 1 - Yes  0 - No |
| **Mechanical ventilation** | 1 - Yes  0 - No |
| **Pulmonary surfactant replacement therapy** | 1 - Yes  0 - No |
| **Complementary exams** |  |
| **Pulmonary X-ray** | 1 - Yes  0 - No |
| **Complete blood count** | 1 - Yes  0 - No |
| **C Reactive Protein (the one with the most altered value)** | nnn.n |
| **Clinical condition** |  |
| **Worst Silverman-Anderson Score (SAS)** | Value |
| **Date** | Day-month-year |
| **Hour** | hour:minute |
| **Neonatal situation at 72 hours of life** |  |
| **Mode of Discharge** | 1 - Alive  2 - Death  3 – Held in the hospital |
| **Discharge date** | Day-month-year |
| **Discharge time** | hour:minute |
| **Date of the neonatal death** | Day-month-year |
| **Time of the neonatal death** | hour:minute |
| **Observations** |  |
